# Global Distribution of *Campylobacter jejuni* Penner Serotypes: A Systematic Review

**DOI:** 10.1371/journal.pone.0067375

**Published:** 2013-06-27

**Authors:** Brian L. Pike, Patricia Guerry, Frédéric Poly

**Affiliations:** Enteric Diseases Department, Infectious Disease Directorate, Naval Medical Research Center, Silver Spring, Maryland, United States of America; The Australian National University, Australia

## Abstract

Penner serotyping has been the principal method for differentiating Campylobacter isolates since its inception. Campylobacter capsule polysaccharide (CPS), the principal serodeterminant on which Penner serotyping is based, is presently of interest as a vaccine component. To determine the required valency of an effective CPS-based vaccine, a comprehensive understanding of CPS distribution is needed. Because of the association between Penner serotype and CPS, we conducted a systematic review to estimate the frequency and distribution of Penner serotypes associated with cases of Campylobacteriosis. In total, more than 21,000 sporadic cases of *C. jejuni* cases were identified for inclusion. While regional variation exists, distribution estimates indicate that eight serotypes accounted for more than half of all sporadic diarrheal cases globally and three serotypes (HS4 complex, HS2, and HS1/44) were dominant inter-regionally as well as globally. Furthermore, a total of 17 different serotypes reached a representation of 2% or greater in at least one of the five regions sampled. While this review is an important first step in defining CPS distribution, these results make it clear that significant gaps remain in our knowledge. Eliminating these gaps will be critical to future vaccine development efforts.

## Introduction

Campylobacter is a gram negative organism that belongs to the class epsilonproteobacteria, and is recognized as a major foodborne pathogen in developed and developing countries alike. Campylobacteriosis, the disease caused by *Campylobacter* spp., is often a self-limiting disease commonly associated with diarrhea, cramping, headache, and fever. However, severe cases, including those resulting in dysentery, may require medical attention. Furthermore, long-term sequelae, including Guillain-Barré Syndrome (GBS), reactive arthritis, and irritable bowel syndrome (IBS) have been linked to infection [1]. The two major *Campylobacter* spp. involved in human diarrheal disease are *C. jejuni* and *C. coli*. However, for the purpose of this systematic review, analysis will be limited to *C. jejuni*.

For the past 30 years, epidemiologic studies of *C. jejuni* have used the Penner serotyping scheme, a passive slide hemaglutination assay developed by Penner and Hennessy [2], to classify differing Campylobacter isolates. This system, which is also called the heat stable or HS serotyping system, was originally thought to be based on lipopolysaccharides (LPS), also referred to as the “O” antigen, hence the frequent designation of “O” serotypes in earlier publications. However, more recent data show that *C. jejuni* does not express LPS, but instead expresses lipooligosaccharides (LOS) and a capsule polysaccharide (CPS) [3], [4]. The CPS has since been shown to be the primary serodeterminant of the Penner scheme [4], [5]. Today, there are 47 recognized HS serotypes of *C. jejuni*, which mirror CPS variation within the species [1]. These 47 serotypes, because of similarities in CPS structure, may further be refined into 35 serotypes, serotype cross-reactive pairs or complexes (See Methods). Because CPS is one of the few identified virulence factors of *C. jejuni* [6] and [7], the potential use of CPS-based vaccines to protect against infection has gained some interest and early evidence appears to support such a strategy. Vaccines based on the CPS of *C. jejuni* strain 81–176 (HS23/36) and CG8486 (HS4 complex) conjugated to CRM_197_ (a diptheria toxin mutant) reduced disease in the intranasal mouse model following challenge with the homologous strain. And, vaccines made with CPS of 81–176 also provided 100% protection against diarrheal disease in *Aotus nancymaae,* a new world monkey, when challenged with 81–176 [8] and 86% protection when challenged with CG8421, another HS23/36 strain which has been used in development of a human challenge model [9] and [Gregory et al. (in preparation)].

These data suggest that CPS based vaccines are a viable strategy to protect against diarrheal disease. However, continued pursuit of such a strategy will require answering questions about the required valency of a broadly effective CPS conjugate vaccine against *C. jejuni*. A first step in that direction will be determining the prevalence of CPS types circulating globally. Due to the correlation between CPS and Penner types, this review aims to summarize Penner serotyping data of clinical isolates, and by extension *C. jejuni* CPS type distribution, as reported in the published literature since the advent of Penner’s method.

## Methods

Relevant published data were identified from searches of PubMed for research articles containing the keyword “Campylobacter” and the term “Penner” or “serotype”. At the same time, non-English publications, and review articles were excluded. The titles and abstracts of the identified articles were screened for relevance and evaluated independently by two of the study authors for inclusion on the basis of the availability of the article, and whether or not the article had previously unpublished, extractable data. Inclusion was limited to studies of natural sporadic *C. jejuni* infections in which human isolates were typed by the Penner-serotyping method. Research articles that reported data on fewer than ten isolates, data from outbreaks, or data from collections of isolates with evidence of selection bias (i.e. studies examining isolates from Guillain-Barré Syndrome patients only) were excluded. No further exclusionary restrictions were applied, such as the makeup of the study population, the length of the observation period, or the publication date. Disagreements between reviewers concerning inclusion were resolved by consensus. Data from studies selected for inclusion were extracted by two authors independently. The data were extracted according to a fixed protocol to include, the author, year, and location of the study, the demographics of the study population, the serotyping methodology used, the length of the study period, and the number of serotypes screened, and a count of the serotypes identified. Extracted data were entered independently into a Microsoft Access (Redmond, WA) database. Reported isolates were assigned to 1 of 35 commonly observed serotypes or cross-reactive serotype groups (HS1/44, HS2, HS3, HS4 complex (includes HS4/13/16/43/50/63/64/65), HS5/31, HS6/7, HS8/17, HS9, HS10, HS11, HS12, HS15, HS18, HS19, HS21, HS22, HS23/36, HS27, HS29, HS32, HS33, HS35, HS37, HS38, HS40, HS41, HS42, HS45, HS52, HS53, HS55, HS57, HS58, HS60, HS62, or Non-Typable). Some Penner serotypes were reported as belonging to more than one of the HS types indicated above, and such isolates were distributed across each serotype indicated. For example, an isolate reported as belonging to serotype HS24/55/60 would be distributed equally as HS24 = 0.33, HS55 = 0.33, and HS60 = 0.33. Several research articles also grouped a fraction of isolates into the non-descript category “Other”, when the relative proportion of a given serotype was below a reporting threshold determined by study authors. In these instances, the serotypes of the “Other” isolates were imputed across each study and distributed in the relative global proportions calculated for the 35 *C. jejuni* serotypes outlined above. *C. coli* serotypes, when reported, were not included in this analysis. Discrepancies concerning serotype assignment were resolved through discussion amongst all study authors. Serotypes were tallied within each study, and their respective proportions were calculated. Pooled proportional estimates were computed across all studies and within studies grouped by region. The proportional estimates were computed using the DerSimonian & Laird random effects model [10]. Strong evidence of heterogeneity existed across the studies for most of the serotypes examined, the exception being those rarely reported in the literature (HS22, HS29, HS32, HS33, HS35, HS38, HS40, HS41, HS42, HS45, HS52, HS55, HS57, HS60, HS62, and HS66). All statistical analyses were performed using Stata Version 12 (College Station, TX).

## Results

A search of the PubMed database identified 596 research articles for possible inclusion. After removing the duplicates, 488 research articles remained for consideration. A review of the titles and abstracts excluded another 410 articles from consideration based on relevance to the topic of interest, leaving 78 studies to be assessed for eligibility for inclusion. The full text of each of the 78 articles was examined in more detail, and data from 54 studies were included for the purpose of this review. Five publications reported stratified data that are included as separate studies for the purpose of this review, bringing the total number of studies to 59 (See Table 1 and Supplementary Figure S1).

**Table 1:** Included Studies.

First Author	Country^a^	Total^b^	Year^c^	Duration^e^	Age^f^	CatchmentArea g, h	Serotypes Tested i
Karmali [13]	Canada	285	1978	36	Children 0 to >10	Point	55
Taylor [14]	USA	46	1980	6	Mixed	Regional	NS
Skirrow [15]	England	3400	1981	132	Mixed	Country	43
McMyne [16]	Canada	153	1982^d^	NS	NS	Regional	55
Lastovica [17]	South Africa	258	1982	6	Children <10	Point	60
Georges-Courbot [18]	CAR	94	1982	17	Children <15	Point	56
Neogi [19]	Bangladesh	102	1983	12	Mixed	Point	42
Patton [20]	USA	149	1985^d^	NS	NS	Country	56
Jones [21]	Britain	406	1985^d^	NS	NS	Unknown	32
Sjogren [22]	Sweden	29	1985	12	Adults >15	Point	23
Sjogren [22]	Mexico	130	1985	12	Infants 0–5	Point	23
Nishimura [23]	Japan	69	1985	NS	NS	Unknown	NS
Chatzipanagiotou [24]	Greece	31	1987	12	Children <14	Point	25
Albert [25]	Australia	108	1988	12	Mixed	Regional	66
Albert [26]	Australia	12	1988	6	Mixed	Regional	66
Sjogren [27]	Kuwait	47	1989 d	NS	Mixed	Point	NS
Zaman [28]	Saudi Arabia	46	1989	12	Mixed	Point	NS
Prasad [29]	India	22	1989	132	Mixed	Regional	72
Wareing [30]	England	754	1990	7	NS	Country	42
Takahashi [31]	Japan	455	1990	156	NS	Country	25
Owen [32]	UK	27	1992	12	NS	Country	45
Asrat [33]	Ethiopia	35	1992	12	Mixed	Point	33
Owen [34]	England	398	1993	12	NS	Country	47
Marshall [35]	England	70	1994 d	NS	NS	Point	NS
Gibson [36]	UK	27	1994	2	NS	Country	45
Nishimura [23]	China	85	1994	NS	NS	Regional	NS
Fang [37]	Taiwan	27	1994	120	Mixed	Unknown	25
Nielsen [38]	Denmark	136	1995	12	NS	Country	49
Nielsen [39]	Denmark	42	1995	11	NS	Country	47
Poly [5]	Egypt	142	1995	43	Infants 0–5	Point	47
Frost [40]	Wales	2310	1996	12	NS	Country	66
Hudson [41]	New Zealand	69	1996	7	NS	Point	NS
Strid [42]	Denmark	173	1996	NS	Mixed	Country	47
Petersen [43]	Denmark	42	1996	24	NS	Country	47
Smith [44]	Nigeria	17	1997 d	NS	NS	Point	64
Sopwith [45]	England	2277	1997	24	Mixed	Regional	NS
McKay [46]	Scotland	3155	1998	12	NS	Country	66
Moser [47]	Germany	201	1998	12	NS	Regional	9
Chatzipanagiotou [24]	Greece	98	1998	24	Children <14	Point	25
Poly [5]	Thailand	103	1998	72	Adults >15	Country	47
Vierikko [48]	Finland	518	1999	3	NS	Country	25
Saito [49]	Japan	158	2000	36	NS	Regional	25
Eyles [50]	New Zealand	54	2000	12	Mixed	Regional	NS
Ioannidis [51]	Greece	207	2000	36	NS	Regional	25
Gilpin [52]	New Zealand	66	2000	6	NS	Regional	NS
Nielsen [53]	Denmark	973	2001	12	NS	Regional	47
Fussing [54]	Denmark	926	2001	13	Mixed	Regional	47
Wierzba [55]	Egypt	20	2001	30	Mixed	Point	NS
Oza [56]	England	414	2002 d	NS	NS	Unknown	66
Cornelius [57]	New Zealand	106	2002	2	NS	Point	NS
Gilpin [58]	New Zealand	168	2002	6	NS	Regional	43
Schonberg-Norio [59]	Finland	114	2002	3	NS	Country	25
Sonnevend [60]	UAE	41	2002	24	NS	Point	25
Nakari [61]	Finland	622	2002	48	Mixed	Country	25
Nakari [61]	Finland	785	2002	48	Mixed	Country	25
Miljkovic-Selimovic [62]	Serbia	29	2003	21	NS	Regional	NS
McTavish [63]	New Zealand	112	2006	NS	Mixed	Country	43
Islam [64]	Bangladesh	31	2006	NS	NS	Point	NS
Grozdanova [65]	Macedonia	20	2008	11	NS	Regional	25

a Country = Country from which sporadic diarrhea cases were identified;

b Total = Total number of isolates analyzed;

c Year = Year specimen collection initiated.

d When the year in which specimen collection began was not specified, publication year used;

e Duration = Length of specimen collection period in months;

f Age in years, “Mixed” indicates specimens collected from both children and adults;

g Catchment indicates the size of the collection area,

h Point = a single collection point (e.g. single hospital or clinic);

i Serotypes Tested = number of serotypes included in the panel of screening sera used in each study. A number of studies screened for *C. coli* serotypes in addition to those for *C. jejuni*. Therefore, the number of serotypes screened for may exceed the 35 *C. jejuni* serotypes enumerated in this review. Abbreviations: CAR = Central African Republic, UAE = United Arab Emirates, UK = United Kingdom, USA = United States of America; NS = Not Specified.

In total, the studies were published between 1982 and 2011, reported data on 21,394 individual *C. jejuni* isolates from sporadic cases of enteric infection collected between 1978 to 2008 from 29 different countries (Table 1). Study size and duration varied considerably. The largest and smallest studies comprised 3,400 and 12 isolates, respectively (mean = 363), while the duration of the studies analyzed ranged from 13 years to 2 months. The included studies also varied in design (i.e. sampling methodology and the size of the catchment area) as well as in their target populations (i.e. age, traveler vs. resident populations). The number of serotypes screened for in each study also differed, ranging from nine to 72 serotypes (including serotypes for *C. coli*) (See Table 1).

**Figure 1:**
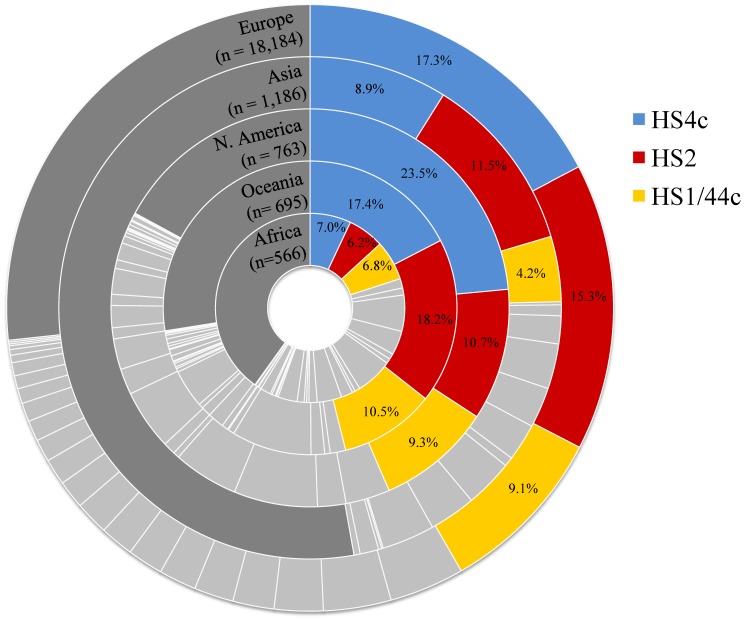
Proportional representation of the three most dominant HS serotypes (HS4c, HS2, and HS1/44) by region. Lightly shaded areas represent the 33 (of 35) HS serotypes not indicated in color on the graph. Darkly shaded areas indicating those isolates not accounted for in the 35 HS serotypes examined were empirically derived by subtracting the sum of the percentages of the 35 serotypes from 100%. The darkly shaded area also includes non-typable isolates.

Overall, the studies predominately sampled European populations. Nearly 85% (n = 18,184) of the isolates included in this analysis were from Europe, while 1,186 were from Asia, 763 were from North America, 695 were from the Oceanic Region, and 566 were from Africa (Figure 1). No studies examining South America were identified in the literature search.

Globally, eight serotypes (HS4 complex, HS2, HS1/44, HS11, HS5/31, HS8/17, HS6/7, and HS3) accounted for 50.4% of all isolates. The dominant serotypes were those of the HS4 complex (15.3%, CI: 12.9, 17.6), HS2 (13.5%, CI: 11.3, 15.8), and HS1/44 (8.2%, CI: 7.1, 9.3) (See Table 2). Combined, these three serotype categories accounted for nearly 40% of all isolates reported worldwide. HS4 complex, HS2, and HS1/44 were also the three serotypes with the greatest proportional representation across each of the five regions examined (Table 3 and Figure 1). Moreover, these three serotypes remained the most prevalent serotypes when the data were stratified by the economic status of the country in which the study was conducted (Tables 4).

**Table 2:** Global HS Serotypes with Proportional Estimates of 2% or Greater.

	%	lci	uci
Global (n = 21,394)			
HS4c	15.3	12.9	17.6
HS2	13.5	11.3	15.8
HS1/44	8.2	7.1	9.3
HS11	3.1	2.2	4.0
HS5/31	2.9	2.2	3.5
HS8/17	2.8	2.2	3.4
HS6/7	2.4	1.8	3.1
HS3	2.2	1.7	2.7

**Table 3:** HS Serotypes with Proportional Estimates of 2% or Greater by Region.

	%	lci	uci
**Africa (n = 566)**			
HS4c	7.0	2.8	11.2
HS1/44	6.8	2.8	10.8
HS3	6.3	1.1	11.6
HS2	6.2	2.1	10.3
HS5/31	6.2	2.3	10
HS23/36	4.2	2.3	6.1
HS8/17	4.1	0.1	8.1
HS53	3.3	0.2	6.4
HS19	2.0	0.6	3.4
**Asia (n = 1,186)**			
HS2	11.5	6.1	17
HS4c	8.9	4.3	13.5
HS1/44	4.2	1.9	6.5
HS15	3.4	1.1	5.7
HS19	3.1	0.9	5.4
HS23/36	3.0	0.9	5.0
HS8/17	2.9	1.0	4.8
HS3	2.6	1.1	4.2
HS37	2.4	0.6	4.1
**Europe (n = 18,184)**			
HS4c	17.3	14.6	20
HS2	15.3	12.1	18.5
HS1/44	9.1	7.7	10.4
HS11	4.0	2.8	5.2
HS6/7	3.6	2.7	4.5
HS5/31	2.6	1.9	3.4
HS8/17	2.2	1.5	2.9
HS12	2.1	1.4	2.8
HS58	2.0	1.0	3.0
**N. America (n = 763)**			
HS4c	23.5	15.3	31.7
HS2	10.7	4.3	17.1
HS1/44	9.3	7.1	11.5
HS5/31	6.8	3.0	10.5
HS8/17	5.3	3.4	7.2
HS3	4.9	1.8	8.1
HS11	3.6	1.2	5.9
HS21	2.5	0.8	4.2
HS6/7	2.3	0.7	3.9
HS18	2.1	0.8	3.4
HS37	2.1	0.7	3.4
**Oceania (n = 695)**			
HS2	18.2	7.9	28.5
HS4c	17.4	10.7	24.0
HS1/44	10.5	6.3	14.8
HS8/17	8.8	3.5	14.1
HS23/36	4.2	2.4	5.9

**Table 4:** HS Serotypes with Proportional Estimates of 2% or Greater by Economic Development Status.

	%	lci	uci
**Developed (n = 1,222)**			
HS4c	17.5	15.2	19.8
HS2	16.5	13.8	19.1
HS1/44	9.0	7.8	10.1
HS11	3.5	2.4	4.5
HS6/7	2.9	2.1	3.6
HS8/17	2.8	2.1	3.4
HS5/31	2.6	2.0	3.3
HS3	2.1	1.6	2.6
**Developing (n = 20,172)**			
HS4c	8.2	4.8	11.5
HS1/44	5.0	2.9	7.1
HS2	5.0	2.8	7.3
HS5/31	4.3	2.3	6.3
HS3	3.7	1.7	5.7
HS8/17	3.5	1.5	5.5
HS23/36	3.3	1.5	5.1
HS15	2.9	1.1	4.6
HS53	2.9	1.0	4.8

Tables 2–4: HS serotypes with a proportional representation of 2% or greater, Globally (Table 2), by Region (Table 3), and by Economic Status (Table 4). Proportional estimates (%) were computed using the DerSimonian & Laird random effects model and include the upper (uci) and lower (lci) 95% confidence intervals. Note: Isolates categorized as a cross-reactive pair HS serotype (e.g. HS1/44, HS5/31, HS6/7, HS8/17, and HS23/36) were originally reported as one of the two serotypes or as the paired serotype itself. Isolates categorized as HS4 complex (or HS4c) represent isolates reported as any combination of the following serotypes HS 4/13/16/43/50/63/64/65.

Beyond the three most dominant serotypes, in all, 17 different serotypes reached a proportional representation of 2% or more in at least one of the five geographic regions considered (Table 5). Nine serotypes reached the 2% threshold in Africa, Asia, and Europe, accounting for 46.1%, 42%, and 58.2% of the total number of isolates in each region, respectively. Combined, the studies from North America had 11 serotypes with a representation of 2% or greater, totaling 73.1% of isolates, while five serotypes met the 2% threshold in Oceania, accounting for 59.1% of isolates in this geographic region. Notably, nearly 14% of isolates were non-typable by the Penner scheme globally (data not shown), a likely consequence of the fact that CPS expression in *C. jejuni* is known to be phase variable [6] and successful typing in the Penner scheme requires CPS expression. As discussed below, methodological differences amongst the studies may also contribute to an artificially inflated number of non-typable isolates.

**Table 5:** Comparison of HS Serotypes with Proportional Estimates by Region: Proportions that met or exceeded the 2% threshold are bolded and those that did not are indicated in italics.

	Global %	Africa %	Asia %	Europe %	N. America %	Oceania %
	(n = 21,394)	(n = 566)	(n = 1,186)	(n = 18,184)	(n = 763)	(n = 695)
HS4c	**15.3**	**7.0**	**8.9**	**17.3**	**23.5**	**17.4**
HS2	**13.5**	**6.2**	**11.5**	**15.3**	**10.7**	**18.2**
HS1/44	**8.2**	**6.8**	**4.2**	**9.1**	**9.3**	**10.5**
HS11	**3.1**	*1.6*	*0.2*	**4.0**	**3.6**	*1.7*
HS5/31	**2.9**	**6.2**	*1.8*	**2.6**	**6.8**	*1.5*
HS8/17	**2.8**	**4.1**	**2.9**	**2.2**	**5.3**	**8.8**
HS6/7	**2.4**	*1.2*	*0.7*	**3.6**	**2.3**	*0.6*
HS3	**2.2**	**6.3**	**2.6**	*1.9*	**4.9**	*0.7*
HS37	*1.8*	*0.9*	**2.4**	*1.8*	**2.1**	*1.8*
HS23/36	*1.7*	**4.2**	**3.0**	*1.4*	*1.8*	**4.2**
HS21	*1.6*	*0.5*	*0.6*	*1.8*	**2.5**	*1.1*
HS19	*1.5*	**2.0**	**3.1**	*1.5*	*0.9*	*0.5*
HS12	*1.3*	*1.0*	*0.0*	**2.1**	*0.5*	*0.7*
HS58	*1.3*	*0.8*	*0.0*	**2.0**	*1.0*	*0.1*
HS15	*1.1*	*1.4*	**3.4**	*1.2*	*0.9*	*0.4*
HS18	*0.9*	*0.4*	*0.1*	*1.1*	**2.1**	*0.2*
HS53	*0.7*	**3.3**	*1.2*	*0.7*	*0.6*	*0.1*

## Discussion

Since Penner first introduced the method [2], serotyping has been an important means of characterizing Campylobacter isolates. Here, using existing data, we estimate the distribution of *C. jejuni* serotypes both globally and by geographic region. Estimates were derived from 59 published studies on more than 21,000 cases of sporadic diarrhea. Based on these estimates, eight serotypes account for half of all isolates globally and three serotypes in particular (HS4 complex, HS2, and HS1/44), were consistently represented across all regions.

Although this study is the first of its kind and a significant step forward in understanding the serotype distribution of *C. jejuni* infections, it is not without limitations. In fact, the estimates presented here are almost certainly imprecise. Data are sparse in every region of the world. No studies reporting extractable data were identified in South America and relatively few studies reported data from Africa and Asia, regions in which enteric infections contribute significantly to morbidity and mortality. The fact that some geographic regions are underrepresented may be partially due to the exclusion of non-English publications. However, the lack of data most probably reflects an absence of surveillance in these regions. With limited data from every region of the world, save Europe, the global estimates presented are biased towards those calculated in Europe. Even in Europe, from which 85% of the isolates in this study originated, there are insufficient data to draw conclusions regarding temporal changes in serotype distribution, geographic variation, and differences across demographic groups (e.g. travelers vs. non-travelers, or children vs. adults, etc.). The estimates presented here are also based on reports of sporadic cases of diarrhea. If an association between serotype and disease severity exists, selection bias has the potential to overestimate serotypes that result in manifest symptoms. Additionally, although a modest number of publications included in this review used a commercially available kit consisting of 25 antisera (Denka Seiken, Co), most studies relied upon custom reagents generated in-house or from another laboratory. The lack of standardized reagents calls into question the comparability of results across individual studies. Similarly, studies varied from one to the next with regards to which and how many serotypes were tested. These methodological differences undoubtedly influenced the estimates calculated here. Studies that did not screen a complete panel of antisera capable of detecting every serotype risked under-reporting certain serotypes, classifying them instead as non-typable. Finally, because *C. jejuni* is known to be subject to phase variation, assays such as Penner serotyping that depend upon the expression of CPS have the potential to underestimate the prevalence of any given *Campylobacter* serotype.

If current efforts to develop a CPS-based vaccine are to succeed, robust surveillance systems are needed to address substantial gaps in knowledge surrounding the geographic distribution and temporal stability of serotypes. Future surveillance methods should also aim to reveal demographic differences in serotype distribution (e.g. age, traveler vs. resident populations) and disease/serotype associations (e.g. severity of disease, risk of developing chronic long-term health outcomes such as reactive arthritis, Guillain-Barré syndrome, or gastrointestinal disorders). Combined with investigations into the immunogenic properties of the differing CPS types, addressing these fundamental surveillance-related questions will be important in determining the composition of a future vaccine with regards to valency. Furthermore, the need for surveillance is greatest in developing regions, where diarrheal disease is most prevalent and available data are lacking. Diarrheal episodes amongst children in the developing world are believed to cause millions of deaths annually [11] and, although the estimates are derived from a relatively small number of studies, the proportion of diarrheal cases attributable to Campylobacter infection is believed to be high, ranging between 5–20% of cases [12]. Given this high incidence rate, the potential benefit of a future vaccine is greatest in the developing world. However, realizing this potential will require a significant surveillance effort to inform the development of a multi-valent vaccine that is well-matched to CPS types circulating in these regions. Implementation of such a surveillance program will require a commitment of time and resources that has not been seen to date. Although Penner typing was once considered the gold standard in *C. jejuni* serotyping, its use has been declining in recent years and, today, the technique is routinely performed by only a small number of reference laboratories in North American and Europe. The limited and declining use of Penner typing is due in part to the complexity and cost of generating polyclonal rabbit sera to the 47 *C. jejuni* type strains, as well as to the emergence and value of other typing schemes such as Multi Locus Sequence Typing (MLST) and the ever-decreasing cost of direct sequencing. For a surveillance system to be implemented that is sufficiently large enough to address the outstanding questions of CPS distribution and disease association, alternative methodologies for determining the CPS type of *C. jejuni* isolates will almost certainly need to be employed. Such alternative methodologies will need to be cost-effective, efficient with respect to time, readily transferred to most any laboratory, and have high throughput capacity. Recently, our group offered a method that meets these criteria. Sequencing has revealed that each Penner serotype is unique with regards to the genomic structure of the cassette of genes involved in the biosynthesis of the serodeterminant CPS [5]. We have designed specific PCR primers that exploit these genomic differences and reproduce the original Penner serotypes. The published system covered 14 serotypes, and has recently been extended to 47 serotypes, (Poly et al. in preparation). Standardization and distribution of this CPS typing system offers one potential alternative method for large-scale surveillance. In addition to the already noted benefits this molecular typing system might offer, such a system may also reduce or eliminate the substantial number of non-typable isolates found in previous studies, as the described PCR-based typing system it is not sensitive to CPS expression. Regardless of which method is ultimately used, informed design of a CPS-based vaccine will require a substantial investment of resources to sustain the intensive surveillance needed to move beyond the incomplete and static picture that this review is able to offer.

## Supporting Information

Figure S1 **Flow diagram of articles search, reviewed, and included in the systematic review.**
(DOCX)
