# Supplementary material for: Global Distribution of Campylobacter jejuni Penner Serotypes: A Systematic Review
Source: PLoS One. 2013 Jun 27;8(6):e67375. doi: 10.1371/journal.pone.0067375 (PMC3694973; doi:10.1371/journal.pone.0067375)
Supplement: Figure S1 — Flow diagram of articles search, reviewed, and included in the systematic review. (DOCX) [file pone.0067375.s001.docx]

**Figure S1: PRISMA 2009 Flow Diagram**

Studies included in qualitative synthesis
(n = 59 ) *Five publications reported stratified data that were included as separate studies for the purpose of this review

Records identified through database searching (PUBMED)
(n = 596 )

Studies included in quantitative synthesis (meta-analysis)
(n = 59 )

Full-text articles excluded, with reasons
(n = 24 )

Full-text articles assessed for eligibility
(n = 78 )

Records excluded
(n = 410 )

Records screened
(n = 488 )

Records after duplicates removed
(n = 488 )

## Identification

## Eligibility

## Included

## Screening
